# Clinical, Biochemical, and Molecular Characterization of Neonatal-Onset Dubin–Johnson Syndrome in a Large Case Series From the Arabs

**DOI:** 10.3389/fped.2021.741835

**Published:** 2021-11-10

**Authors:** Abdulrahman Al-Hussaini, Badr AlSaleem, Hamad AlHomaidani, Ali Asery, Muhanad Alruwaithi, Mohammed Alameer, Waleed Afashah, Bashir Muhammed Salman, Naif Almontashiri

**Affiliations:** ^1^The Division of Pediatric Gastroenterology, Children's Specialized Hospital, King Fahad Medical City, Riyadh, Saudi Arabia; ^2^College of Medicine, Alfaisal University, Riyadh, Saudi Arabia; ^3^Prince Abdullah bin Khalid Celiac Disease Research Chair, Department of Pediatrics, Faculty of Medicine, King Saud University, Riyadh, Saudi Arabia; ^4^Department of Molecular Genetics, Children's Specialized Hospital, King Fahad Medical City, Riyadh, Saudi Arabia; ^5^Department of Biostatistics, Research Services Administration, Children's Specialized Hospital, King Fahad Medical City, Riyadh, Saudi Arabia; ^6^Center for Genetics and Inherited Diseases, Taibah University, Medina, Saudi Arabia; ^7^Faculty of Applied Medical Sciences, Taibah University, Medina, Saudi Arabia

**Keywords:** Dubin-Johnson syndrome, *ABCC2* gene, Saudi Arabia, neonatal cholestasis, direct hyperbilirubinemia, normal alanine aminotransferase

## Abstract

**Background:** There are only a few case reports and small case series on neonatal-onset Dubin–Johnson syndrome (DJS), particularly from Far-East Asia, Iranian and Moroccan Jews, and Europe.

**Objectives:** In this first study from the Arabs and the largest series reported to date, we characterized the clinical, laboratory, and molecular features and outcome of gene-confirmed neonatal-onset DJS.

**Methods:** We reviewed our database of 533 cases of neonatal cholestasis that presented to our center during the period from 2008 to 2019. We identified neonates with a disease-causing mutation in *ABCC2* gene.

**Results:** Twenty-eight neonates with DJS were diagnosed (5.3%). All of the 28 were full-term, well looking neonates without hepatosplenomegaly, with cholestasis, and normal liver synthetic function since the 1 week of life that resolved within 3–6 months of age, followed by a benign course punctuated by recurrent episodes of jaundice in 43% during a median follow up period of 9.25 (range 2.5–14 years). Alanine aminotransferase levels were within normal range in 26 patients (92%) and mildly elevated in two patients. ALT levels were significantly lower in neonates with DJS than in other cases with neonatal cholestasis from other causes (*p* < 0.001). The median urinary coproporphyrin I% was 88% (IQ1–IQ3 = 84.2–92.7%). We identified four homozygous variants in the *ABCC2* gene (from 22 unrelated families), one splicing variant (c.3258+1G>A; p.?), and three were missense variants; two of which were novel missense variants [c.1594G>A (p.Glu532Lys) and c.2439G>C (p.Lys813Asn)]. The p.Gly758Val mutation has occurred in 23 patients (from 19 unrelated families).

**Conclusions:** Our study suggests that normal ALT-cholestasis in a well-looking neonate should trigger evaluation for DJS. The p.Gly758Val variant in ABCC2 is the most predominant mutation among Arabs with “founder effects.” Identification of the predominant *ABCC2* variant in any population is likely to facilitate rapid molecular analysis by future targeting of that specific mutation.

## Introduction

Dubin–Johnson syndrome (DJS) is a rare cause of neonatal cholestasis. It results from variants in the ATP-binding cassette-subfamily C member 2 (*ABCC2)* gene leading to absent, reduced expression, or impaired function of a transporter protein known as multidrug resistance-associated protein 2 (MRP2) (1). The MRP2, which is located on the canalicular membrane of hepatocytes, is important in excretion of conjugated bilirubin from hepatocytes into the bile (2). Since molecular characterization of the first *ABCC2* gene variant responsible for DJS in 1997 (1), several variants of *ABCC2* gene were identified in DJS patients from different populations and ethnicities, particularly from Far-East Asia (Japan, Taiwan, Korea, and China), Israel (Iranian and Moroccan Jews), and Europe (3–14). Most of these studies included children and adults (3–8), however neonatal-onset DJS was rarely reported, and the reports constituted only few case reports and small case series (9–14).

Data on DJS among the Arabs is still lacking, which prompted us to present the largest case series ever reported of 28 neonatal-onset DJS patients with confirmed *ABCC2* gene variants. The objectives of our study were to determine the prevalence of DJS as a cause of neonatal cholestasis (NC), and characterize the epidemiological, clinical, laboratory, and molecular features and outcome of gene-confirmed neonatal-onset DJS among the Arabs as compared with other ethnicities.

## Patients and Methods

### Study Settings and Design

We retrospectively reviewed our database of 533 cases of NC, defined clinically as the presence of jaundice with a conjugated bilirubin of >17 μmol/L, that presented to our center, a tertiary care, non-transplant center for children with liver disorders in Saudi Arabia, during the period from 2008 to 2019. We identified and extracted data for neonates with disease-causing variants in the *ABCC2* gene.

### Hospital Protocol

All neonates/infants presenting to our center with cholestasis undergo extensive work up to exclude infectious, structural, metabolic, endocrine, infiltrative, and familial causes. In our protocol, we considered liver biopsy when biliary atresia (BA) is highly suspected [high gamma-glutamyl transferase (GGT) cholestasis and pale stool]. In the initial stage of the work up, we have looked carefully to promptly diagnose and treat treatable disorders. When diagnosis remained undetermined after the initial extensive investigations, infants underwent molecular analysis using targeted gene sequencing, if the phenotype of the patient was consistent with a specific genetic disease, or cholestasis panel. After collection of serum for bile acid analysis, all cases were treated with ursodeoxycholic acid (Dr. Falk, Germany) (20 mg/kg/day, in divided doses twice a day) and supplied with fat-soluble vitamins.

### Study Procedures

#### Data Collection

Medical records were reviewed to collect (1) demographics and clinical characteristics, (2) laboratory investigations at presentation: total and direct bilirubin (TSB/DB) (normal reference was 3.4–17.1 μmol/L and 0–6.8 μmol/L, respectively), alanine transaminase (ALT), aspartate transaminase (AST), INR, gamma-glutamyl transferase (GGT), and serum total bile acids (normal, 0–10 μmol/L), (3) urinary coproporphyrins (UCP) levels, (4) imaging findings (ultrasound of abdomen and hepatic scintigraphy), (5) histopathological findings, and (6) outcomes including resolution of cholestasis, defined as TSB level <20 μmol/L, and recurrence of jaundice later on in life. During infancy, the age-specific normal GGT reference range were as following: <1 month old, GGT < 200 IUL; 1–2 months old, GGT < 150 IU/L; 2–3 months old, GGT < 100 IU/L; >6 months, GGT < 60 IU/L (15, 16). Our laboratory used different ALT and AST tests during the study period with different normal cut off reference ranges (Table 1), therefore in addition to reporting the absolute values of the transaminases, we calculated the proportion of ALT and AST to the upper limit of normal (ULN). Several previous studies on DJS patients showed that serum ALT levels were typically normal (9–14). To evaluate the utility of serum ALT level as a biochemical discriminative indicator of DJS, we compared the ALT values in DJS patients with ALT values in other major etiologies contributing to infantile cholestasis in Saudi Arabia. Idiopathic neonatal hepatitis defined a syndrome where extensive investigations into infectious, structural, metabolic, endocrine, infiltrative, and familial causes of cholestasis failed to provide an explanation. The diagnoses of all hereditary diseases were confirmed by molecular analysis. All infants with inborn errors of metabolism and characteristic pattern of metabolites on metabolic screening tests were confirmed by molecular genetic diagnosis.

**Table 1:** Clinical, laboratory, and molecular characteristics and outcome of the 28 patients with neonatal-onset Dubin–Johnson syndrome.

**Labs at presentation**														
**Patient**	**Sex**	**Family (no.)**	**Family history of jaundice**	**Age at onset of jaundice (weeks)**	**Age at presentation (weeks)**	**TSB/DB (μmol/L)**	**ALT/AST IU/L proportion to ULN^‡^**	GGT IU/l^*^	**S. bile acid μmol/L**	**UCP 1%**	**Molecular test used**	**TSB/DB at last FU**	**Recurrence of jaundice (yes/no)**	**Last follow up age (year)**
1	F	A1	Yes	1	5	110/94	31/39 0.56/1.1	145	90	88%	Cholestasis panel^*^	44/29	Yes	8.5
2	F	A2	Yes	1	4	185/159	22/28 0.4/0.8	195	130	92%	Target variant sequence	23/12	No	5
3	F	B1	No	1	2	131/125	33/43 1/1.3	33	155	94%	*ABCC2* sequence	NA	NA	7 Lost to FU
4	F	C1	NA	1	6	140/120	39/44 0.6/1.2	90	177	85%	*ABCC2* sequence	11/9	No	10
5	M	D1	Yes	1	4	216/173	35/62 0.5/1.6	304	158	88%	*ABCC2* sequence	25/20	No	11.5
6	M	E1	NA	1	4	118/102	65/72 **1.6**/1.7	55	70	84%	*ABCC2* sequence	19/16	No	7
7	M	F1	Yes	1	8	174/162	25/32 0.6/0.8	130	48	89%	*ABCC2* sequence	17/14	No	8.5
8	M	G1	No	1	16	42/31	22/34 0.4/1	22	36	82%	*ABCC2* sequence	12/9	Yes	3
9	F	H1	No	1	3	55/46	15/26 0.3/0.8	69	77	92%	*ABCC2* sequence	15/11	No	4
10	F	I1	Yes	1	3	173/140	64/73 1/2	183	117	95%	*ABCC2* sequence	21/15	No	13
11	F	J1	Yes	1	5	137/111	23/18 0.35/0.5	131	156	86%	Target variant sequence	40/25	Yes	13
12	M	J2	Yes	1	7	89/78	33/48 0.8/1.2	149	57	82%	*ABCC2* sequence	82/60	Yes	14
13	M	K1	Yes	1	2	355/270	36/34 0.9/0.9	207	177	94%	*ABCC2* sequence	NA	No	5.5 Lost to FU
14	F	L1	No	1	2	707/470	14/33 0.2/0.9	148	28	86%	*ABCC2* sequence	24/16	No	10
15	M	M1	NA	1	5	173/169	47/66 1.1/1.7	68	180	89%	WES	15/13	No	6
16	F	N1	Yes	1	6	150/139	28/44 0.5/1.3	190	110	82%	WES	56/39	Yes	13
17	M	N2	Yes	1	4	240/218	41/46 0.74/1.3	220	174	88%	Target variant sequence	52/35	Yes	15
18	M	O1	No	1	3	446/314	14/35 0.3/1	295	106	90%	*ABCC2* sequence	19/15	Yes	2.5
19	M	P1	Yes	1	12	93/77	32/38 0.5/1	113	ND	82%	Target variant sequence	22/17	NA	13.5
20	F	P2	Yes	1	7	145/108	22/42 0.3/1.1	159	157	84%	*ABCC2* sequence	20/18	NA	10
21	F	Q1	Yes	1	5	181/172	27/37 0.85/1.1	117	88	96%	WES	30/21	Yes	6
22	M	R1	No	1	18	22/20	34/20 0.5/0.54	20	ND	97%	*ABCC2* sequence	18/13	Yes	10.5
23	F	S1	Yes	1	2	186/167	18/25 0.6/0.8	87	37	88%	*ABCC2* sequence	23/19	NA	8
24	M	T1	Yes	1	6	358/292	34/77 0.5/2	282	105	91%	*ABCC2* sequence	14/11	No	11
25	F	T2	Yes	1	11	257/232	81/88 **2**/2.7	292	ND	85%	Target variant sequence	21/17	No	5
26	M	U1	Yes	1	4	212/97	26/46 0.4/1.2	100	72	95%	WES	25/21	Yes	10
27	F	U2	Yes	1	8	160/153	20/38 0.64/1.2	179	49	93%	*ABCC2* sequence	14/10	Yes	6.5
28	F	V1	No	1	5	178/158	38/44 0.7/1.3	220	ND	84%	*ABCC2* sequence	52/40	Yes	11.5

*TSB/DB, total serum bilirubin/direct bilirubin; ALT, alanine aminotransaminase; F/U, follow up; GGT, gamma glutamyl transferase; M, male; F, female, ND, not done; NA,not available;*

* *normal values of GGT are as the following: <1 month old, GGT < 200 IU/L; 1–2 months old, GGT < 150 IU/L; 2–3 months old, GGT < 100 IU/L; > 6 months, GGT < 60 IU/L.16,17;*

‡ *Our laboratory used different ALT and AST tests during the study period with different normal cut off reference ranges, therefore in addition to reporting the absolute values of the transaminases; we calculated the proportion of ALT and AST to the upper limit of normal (ULN)*.

* *Cholestasis panel = ABCB11, ABCB4, ABCC2, AKR1D1, ATP8B1, BAAT, CLDN1, HSD3B7, JAG1, NOTCH2, NR1H4, SERPINA1, SLC25A13, TJP2, VIPAS39, and VPS33B*.

*The bolded values indicate the 2 patients with slightly elevated ALT calculated to the upper limit of normal (ULN)*.

#### Hepatic Scintigraphy

Technetium-99 m bromo trimethyl-imminodiacetic acid (1–2 mCi) was injected intravenously, and the dynamic images over the abdomen were obtained in anterior and posterior projections for 1 h post injection of the pharmaceutical. Delayed static imaging data were also obtained after 2, 4, and 24 h. A normal study defined prompt hepatic uptake of the tracer followed by excretion in the gut and visualization of the gallbladder and common bile ducts within 10–20 min after injection of the tracer.

#### Urinary Coproporphyrins Analysis

During admission, 24-h urine samples were obtained by urine bags and collected in a container to which sodium carbonate was added to convert all coproporphyrins to their oxidized forms. Urinary coproporphyrins levels were determined by high-performance liquid chromatography in the Mayo Clinic Laboratory. The UCP I% was obtained by dividing the peak height (mV) of isomer I by the sum of peak heights of isomers I and III, and multiplying by 100 to obtain a percentage.

#### Molecular Studies and Variant Interpretation

Throughout the study period, DNA from blood samples was subjected to targeted sequencing, next-generation sequencing (Jaundice chip or cholestasis panel), or whole-exome sequencing (WES). Variants in the *ABCC2* gene were classified according to the classification guidelines of the American College of Medical Genetics and Genomics. Parental testing for the familial variant was performed by Sanger sequencing whenever possible, particularly when the detected *ABCC2* variant is novel.

### Ethics

This study was approved and performed under the ethical guidelines issued by our institution (IRB number 14-012, January 16, 2014), with written informed consent obtained from the parents to do the molecular gene testing.

### Statistical Analysis

All categorical variables such as gender, family history of jaundice, recurrence of jaundice, etc., are presented as numbers and percentages. Continuous variables such as serum ALT levels are expressed as median and interquartile range (IQR). Non-parametric tests were used when data were skewed. Kolmogrov–Smirnov test was used to check the assumption of normal distribution. Box-whisker plot was created to check the distribution of data set and divided into five-point summary theory “minimum,” first quartile (Q1), median, third quartile (Q3), and “maximum.” Chi-square/Fisher's exact test was used according to whether the cell expected frequency is <5, and it was applied to determine the significant association between categorical variables. To evaluate the utility of normal serum ALT level in the diagnostic approach of neonates with cholestasis, the serum ALT levels of neonates with DJS and other neonates/infants with cholestasis from other causes were compared using the Kruskal–Wallis test. A two-tailed *p* < 0.05 was considered as statistically significant. All data was entered and analyzed through statistical package SPSS 25 (*SPSS Inc., Chicago, IL, USA*) and MEDCALC version 18.11.6 (*Acacialaan 22 8400 Ostend Belgium*).

## Results

### Clinical and Laboratory Characteristics of the Neonatal-Onset Dubin–Johnson Syndrome Patients

From 2008 to 2019, 22 unrelated Saudi families with a total of 28 cases with NC were diagnosed with DJS, thus DJS accounted for 5.3% (28/533) of the causes of infantile cholestasis in our center. Table 1 summarizes the clinical and laboratory characteristics and outcomes of the 28 cases. Consanguinity was reported in all the 22 families; 12 of the families (55%) reported history of recurrent jaundice in at least one close relative. All patients were products of full-term pregnancy with a median birth weight of 2.7 kg (range 2.3–4.5 kg) with onset of jaundice within the 1 week of life. They presented to our center at a median age of 5 weeks (range 2–18 weeks). All were clinically well and thriving at the time of presentation. Stool was acholic in three and pigmented in the remaining 25 neonates. Twelve of the 22 families reported a history of recurrent jaundice in close relatives (55%).

**Figure 1:**
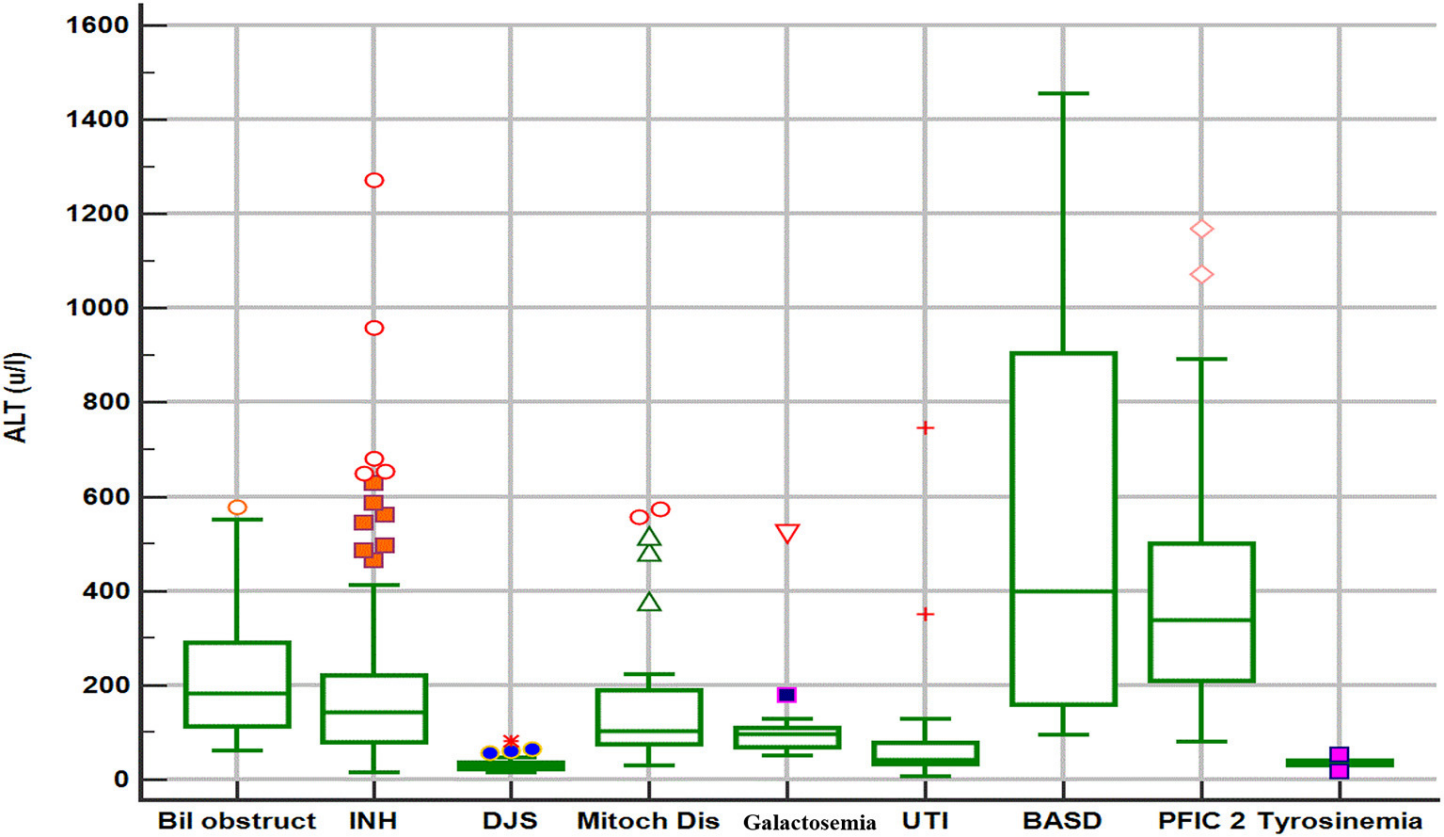
Box plot graph of alanine aminotransferase values in different etiologies of neonatal cholestasis. Bil obstruct, biliary obstruction (biliary atresia and choledochal cyst); DJS, Dubin–Johnson syndrome; INH, idiopathic neonatal hepatitis; Mitoch Dis, mitochondrial disorders; PFIC2, progressive familial intrahepatic cholestasis type 2; BASD, bile acid synthetic disorders; UTI, urinary tract infection.

The biochemical profile at presentation was characterized by normal liver synthetic function and high serum bile acids in all patients, high GGT-cholestasis in 11 (39%), normal GGT in 14 (50%), and low GGT in 3 (11%). ALT level was within normal range in 26 patients (92%) and mildly elevated in two patients (1.6 × the ULN in patient E1 and 2 × ULN in patient T2). The median of UCP I% was 88%, (IQ1–IQ3 = 84.2–92.7%). The box plot graph in Figure 1 shows the statistically significant lower median [interquartile range (Q1–Q3)] ALT level in the DJS group [28.5 (22.7–36.7 IU/L)] as compared with the other causes of neonatal cholestasis in our center: biliary obstruction [*n* = 37; 182 IU/L (113–293), *p* < 0.001]; idiopathic neonatal hepatitis [*n* = 150; 142 IU/L (79–221), *p* < 0.001]; mitochondrial hepatopathy [*n* = 27; 102 IU/L (73–190), *p* < 0.001]; galactosemia [*n* = 13; 96 IU/L (65–116), *p* = 0.002]; urinary tract infection [*n* = 17; 43 IU/L (33–79), *p* < 0.012]; bile acid synthesis disorder [*n* = 17; 399 IU/L (141–943), *p* = 0.002]; and progressive familial intrahepatic cholestasis type two [*n* = 31; 337 IU/L (206–516), *p* < 0.001]. The only exception was tyrosinemia (*n* = 6) with median ALT level of 32 (27–42 IU/L, *p* = 0.75). Ultrasound of the abdomen revealed normal sized-liver and spleen in all patients.

The jaundice resolved in all patients within the first 3–6 months of life. Ursodeoxycholic acid was prescribed in all patients for 3–4 months. All the 28 patients are alive and well after a median follow-up period of 9.25 years (range 2.5–14 years). During the follow-up period, all patients had persistent mild elevation of TSB/DB on repeated liver function tests, but 12 developed recurrent clinically visible jaundice (43%).

### Hepatic Scintigraphy Findings

The hepatobiliary iminodiacetic acid (HIDA) scan was performed in 11 patients. In four patients (A2, I1, O1, and S1), the HIDA scan showed no excretion of the tracer into the gut and non-visualization of the gallbladder after 24 h. In two patients (J1 and T1), the HIDA scan showed significantly delayed excretion of tracer into the gut and visualization of the gallbladder only on the 24 h images of the study. In another four patients (J2, P1, P2, and T2), the excretion of tracer into the gut and visualization of the gallbladder were observed at 1–2 h of the study. In one patient (M1), the study was normal.

### Liver Histopathology

**Figure 2:**
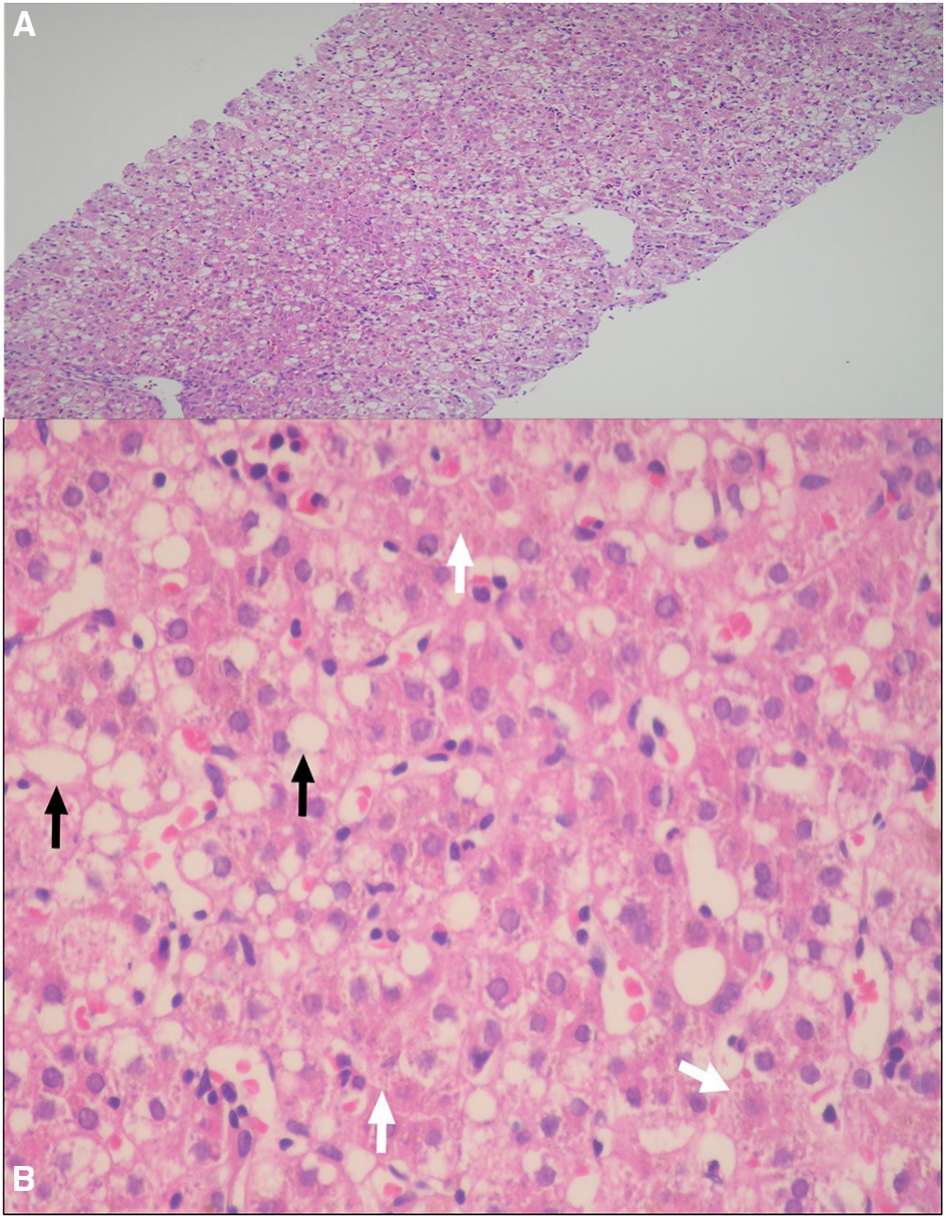
**(A,B)** Percutaneous liver biopsy demonstrating: **(A)** Marked micro- and macrosteatosis of hepatocytes [hematoxylin and eosin (HE), original magnification ×4]; **(B)** Dark pigment granules in hepatocytes (arrows heads), and steatosis of hepatocytes (arrow) (HE 20×).

Percutaneous liver biopsy was performed in two patients. Patient I1, presented with acholic stool, high GGT cholestasis, and non-excretory HIDA scan, and because of the suspicion of BA, the patient underwent wedge liver biopsy and intraoperative cholangiogram, which revealed patent common bile duct. The liver biopsy showed canalicular cholestasis and mild portal fibrosis but no evidence of dark-pigment granules in hepatocytes nor giant cell transformation or ballooning of hepatocytes. Patient D1, had a liver biopsy done in the referring hospital because of the suspicion of BA and the liver histopathology showed micro- and macrosteatosis and dark-pigment granules in hepatocytes (Figures 2A,B).

### Molecular Analysis

The molecular genetic features of this cohort are summarized in Table 2. We identified four homozygous variants in the *ABCC2* gene, one was splicing (c.3258+1G>A; p.?) and three were missense variants; two of which (in two siblings T1 and 2, and V1) were novel missense variants [c.1594G>A (p.Glu532Lys); c.2439G>C (p.Lys813Asn)]. Three of the four variants were classified as pathogenic or likely pathogenic as per the published guidelines of the American College of Medical Genetics and Genomics. The most common variant was c.2273G>T (p.Gly758Val); which was detected in 23 patients (82%) from the eight major tribes in Saudi Arabia and included 19 unrelated families (19/22 families, 86%) (Families A1 to S1). Five of the eight tribes were from the central region (A1 to M2, P1 and 2, and R1), two were from the western region (N1 and 2, O1, and Q1), and one from the southern region (S1).

**Table 2:** The four detected disease-causing variants in the *ABCC2* gene.

**Family ID**	**No. of patients**	***ABCC2* variant**	**Zygocity**	**Type**	**Frequency in SHGP**	**Frequency in gnomAD**	**Classification** **(ACMG guidelines)**	**Variant novelty**
A1–S1	23	NM_000392.5: c.2273G>T; p.Gly758Val	Homozygous	Missense	15/2,936	1/251,268	Pathogenic	Reported (PMID: 31544333)
T1 and T2	Two siblings	NM_000392.5: c.1594G>A; p.Glu532Lys	Homozygous	Missense	Absent	Absent	Likely pathogenic	Novel
U1 and U2	Two siblings	NM_000392.5: c.3258+1G>A; p.?	Homozygous	Splicing	1/2,936	21/282,862	Pathogenic	Reported (PMID: 23429660)
V1	One	NM_000392.5: c.2439G>C; p.Lys813Asn	Homozygous	Missense	Absent	Absent	Likely pathogenic	Novel

*ACMG, American Academy of Medical Genetics; genomAD, Genome Aggregation Database; SHGP, Saudi Human Genome Project*.

## Discussion

This is the first study from the Arabs and the largest series reported to date to describe the clinical and biochemical features and molecular landscape of 28 neonatal-onset DJS patients. Our study highlights multiple important observations. First, DJS accounted for 5.3% of the causes of infantile cholestasis in our Saudi population, which is similar to the prevalence of BA in the same population (5%) (17). Second, the typical patients with neonatal-onset DJS are likely to be full-term, well looking neonates who manifest with normal ALT cholestasis since the 1 week of life, which resolves within 3–6 months of age, followed by a benign course that could be punctuated by recurrent episodes of jaundice on long term follow-up (in some patients), and a direct bilirubin that do not normalize between episodes. Third, the lack of dark-pigment granules in hepatocytes, normal hepatic HIDA scan, or mildly elevated liver transaminases do not exclude the diagnosis of DJS in neonates. Another important finding in our cohort is the identification of the recurring homozygous *ABCC2* gene variant [c.2273G>T (p.Gly758Val)] in 19 unrelated families from eight tribes in Saudi Arabia, that has not previously been reported in other populations; highly suggesting that this variant came from a common ancestor, i.e., “founder in nature.”

Upon reviewing the reported cases, the clinical presentation of DJS as NC is rare, however, our study shows that DJS represents an important disease to consider in the differential diagnosis of NC among the Saudi Arabian population similar to the high frequency reported in the Far-East Asian populations (9–11, 18). The clinical phenotype and biochemical profile (normal or elevated GGT, and high total serum bile acids) of neonatal-onset DJS overlap with a broad list of causes of NC, which makes the identification of DJS challenging to clinicians. Notably, some neonates with DJS present with acholic stool and high-GGT cholestasis, a phenotype similar to neonates with BA, and might undergo invasive procedures (liver biopsy and intraoperative cholangiography), a scenario that happened frequently in some reported case series (9, 10, 19) and in three of our patients as well. Hence, early consideration and prompt diagnosis of DJS is a very important step in the work up of a neonate with cholestasis to avoid subjecting a patient with a benign prognosis to unnecessary invasive and costly evaluation. In contrast to the vast majority of causes of NC, DJS is not associated with obvious liver injury, as evident by normal ALT, and absence of hepatosplenomegaly. Indeed, as shown in Figure 1, our data support that normal ALT level is a very important biochemical discriminative marker that differentiates DJS from other major causes of NC in our population and useful to direct the investigations toward DJS. However, our data suggest that mildly elevated ALT (not >2× ULN) should not exclude DJS diagnosis. Other differential diagnosis of normal ALT-cholestasis in our cohort of 533 cases of NC (unpublished data) included urinary tract infection, sepsis, inspissated bile due to hemolysis, tyrosinemia, parenteral nutrition-associated cholestasis, and few cases of idiopathic neonatal hepatitis. These diagnoses are readily identifiable by following a structured, stepwise diagnostic approach that incorporates clinical assessment and laboratory investigations (e.g., urine and blood cultures, complete blood count and reticulocytes, and metabolic workup) as appropriate. Hence, a well-looking neonate with normal ALT-cholestasis, no hepatosplenomegaly, normal liver synthetic function, and no evidence of infection or hemolysis, should raise suspicion and prompt evaluation for DJS.

In practice, when the abovementioned DJS phenotype is suspected, and before performing a confirmatory molecular analysis of the *ABCC2* gene, the next step in evaluation depends on the diagnostic modalities available in every hospital. For example, UCPA, available in very few centers, is the most helpful non-invasive test with a very high diagnostic performance. Our data and others (14, 19) suggest that a percentage of UCP isomer I > 80% of the total UCPs would be consistent with a diagnosis of DJS. HIDA scan is another useful non-invasive tool to evaluate and orient the diagnosis toward DJS that it is more readily available than UCPA, however is less sensitive and specific than UCPA. On HIDA scan, patients with DJS typically show prompt hepatic uptake of the radioisotope, nonvisualization of the gallbladder, and delayed or non-excretion in the gut (20); such characteristic pattern in a patient with DJS phenotype should therefore alert clinicians to the strong possibility of DJS and prompts a confirmatory gene testing. On the other hand, normal HIDA scan, as observed in one of our 11 patients in whom the study was performed, does not exclude DJS diagnosis. The third tool that was frequently employed to diagnose DJS phenotype, before the first molecular characterization of *ABCC2* gene in 1997 (1), is liver biopsy. The characteristic histopathological finding of melanin-like pigment deposits in hepatocytes is uncommon in neonates compared with adults (9, 10). Thus, the low diagnostic yield and invasiveness of the procedure make liver biopsy an unfavorable diagnostic tool of neonatal-onset DJS, particularly in the era of the advanced molecular genetics and availability of non-invasive methods. Of note, the hepatic steatosis observed in one of our patients, was also reported in five neonatal-onset DJS patients from Japan (9, 21). Therefore, DJS should be added to the list of differential diagnoses of NC associated with hepatic steatosis (22).

The mutational spectrum of the *ABCC2* gene in DJS patients includes more than 60 different variants, described from different populations (3–14), that are predicted to cause defects in the protein structure, maturation, localization to the canalicular membrane, or the function of correctly localized protein (23). These variants vary across different ethnic groups. The p.R768W variant occurred with a high frequency in the Japanese population (9), while the p.Arg768Trp is the most common variant in the Korean population (10), and the p.R393W and p.Y1275X variants are known disease-causing variants in Taiwan (11). Some variants are unique to the Jewish population of Iranian and Moroccan origins (p.I1173F and p.R1150H, respectively) (3). Our molecular analysis of the *ABCC2* gene showed that the p.Gly758Val variant is the most common variant in the Saudi patients, affecting 82% of the patients with DJS in this study. This variant is unique to the Saudi population and found in several major tribes in the center of the Arabian Peninsula with a cumulative carrier frequency of 0.0051 (one in 195), strongly suggesting that this variant is of common ancestral origin, which accounted for the observed cluster. Two of the four *ABCC2* gene variants identified in two families from our study cohort [c.3258+1G>A and c.2439G>C (p.Lys813Asn)] were previously reported in the Asian populations (9, 10). The prevalence of consanguineous marriage in Saudi Arabia is almost 60% (24), which presents a major risk factor for autosomal recessive diseases.

The correlation between specific pathogenic variants of *ABCC2* and DJS phenotype (particularly as related to age of onset, neonatal vs. adult onset DJS) has been investigated in a few studies. Some investigators reported that variants involving the ATP-binding cassettes (ABC) of MRP2 protein and those resulting in severely dysfunctional or absent protein (like splicing, truncating, and frameshift variants) were more common in patients with neonatal-onset DJS, whereas variants involving domains other than the ABC of the MRP2 protein were commonly observed in adults (9, 11). According to our data, only two of the four variants identified involve the ABC region of the protein (p.Glu532Lys and c.3258+1G>A) and three are homozygous missense variants (Table 2). However, two of the three missense variants (p.Gly758Val and p.Lys813Asn) are located in a highly conserved amino acid domain in close proximity to the highly conserved acceptor splice site in exons 12 and 18, respectively.

Besides the retrospective design of the study with its inherent limitations, we did not perform immunohistochemical staining of liver biopsies for MRP2 in these patients, hence we could not determine the level of expression and loss of function of the protein caused by these variants. Therefore, further genetic and functional studies of neonatal-onset DJS are needed to determine the genotype-phenotype correlation.

## Conclusions

We found that normal ALT level and UCP I > 80% in a neonate with cholestasis are very important biochemical markers that characterize DJS. Mutation analysis confirms that the most common and founder mutation in the Saudi patients with neonatal-onset DJS is p.Gly758Val variant in *ABCC2*. Identification of the predominant *ABCC2* variant in any population is likely to facilitate rapid molecular analysis by future targeting of that specific mutation.

## Data Availability Statement

The original contributions presented in the study are included in the article/supplementary material, further inquiries can be directed to the corresponding author.

## Ethics Statement

The studies involving human participants were reviewed and approved by the local review board in King Fahad Medical City approved the study (IRB number 14-012). The patients/participants provided their written informed consent to participate in this study.

## Author Contributions

AA-H contributed to the study conception and design, acquisition of the data, and writing of the manuscript. HA and NA analyzed and interpreted the results of the gene tests. BA, AA, and MAlr provided clinical advice. MAla and WA collected and analyzed the data. BS performed the statistical analysis. HA, BA, AA, MAlr, MAla, WA, BS, and NA reviewed and approved the manuscript. All authors contributed to the article and approved the submitted version.

## Funding

The authors acknowledge the financial support from Prince Abdullah bin Khalid Celiac Disease Research Chair, Department of Pediatrics, Faculty of Medicine, King Saud University.

## Conflict of Interest

The authors declare that the research was conducted in the absence of any commercial or financial relationships that could be construed as a potential conflict of interest.

## Publisher's Note

All claims expressed in this article are solely those of the authors and do not necessarily represent those of their affiliated organizations, or those of the publisher, the editors and the reviewers. Any product that may be evaluated in this article, or claim that may be made by its manufacturer, is not guaranteed or endorsed by the publisher.
